# Muddles and puzzles: Metaphor use associated with disease progression in Primary Progressive Aphasia

**DOI:** 10.1080/02687038.2023.2257356

**Published:** 2023-10-16

**Authors:** Anna Volkmer, Jade Cartwright, Leanne Ruggero, Maria Loizidou, Chris JD Hardy, Deborah Hersh

**Affiliations:** a Psychology and Language Sciences, University College London, UK; b School of Health Sciences, University of Tasmania, Launceston, Australia; c School of Psychological Sciences, Macquarie University, Sydney, Australia; d Department of Neurodegenerative Disease, Dementia Research Centre, UCL Queen Square Institute of Neurology, University College London, London, UK; e Curtin School of Allied Health, and Curtin enAble Institute, Curtin University, Perth, Australia

**Keywords:** Primary progressive aphasia, dementia, language, speech and language therapy, Metaphor

## Abstract

**Background:**

Primary Progressive Aphasia describes a language-led dementia and its variants. There is little research exploring the experiences of living with this disease. Metaphor, words that represent something else, have been studied extensively in health-related narratives to gain a more intimate insight into health experiences.

**Aims:**

This study explored the metaphors used spontaneously by people with PPA, their care partners (family), and speech and language therapists/pathologists (SLT/Ps) providing support along the continuum of care.

**Methods & Procedures:**

This study examined two previously collected data sets comprising naturalistic talk where metaphors were not the specific focus, the first from focus groups conducted with people with PPA and their families and the second from focus groups conducted with SLT/Ps working with people with PPA. Transcribed data were analysed for metaphor use through an iterative narrative approach.

**Outcomes & Results:**

In all, 237 examples of metaphorical language were identified in the data, with 14 metaphors from people with PPA, 116 from the families and 106 from SLT/Ps. Different metaphors were used by participants to describe their experiences depending on which variant of PPA they were living with, and people also described their disease differently over time. SLT/Ps also used metaphors, however, their language reflected the structured, professional perspective of delivering speech and language therapy services.

**Conclusions & Implications:**

SLT/Ps should listen for and recognise the metaphorical language used by people with PPA and their families to ensure therapeutic alignment, see beyond the PPA to recognise the individual’s needs, and provide person-centred and empathic support.

## Background

**Table:** 

Case example: A client is attending their appointment in the multidisciplinary memory disorders clinic. When the consultant neurologist asks, “and how have you been?”, the client responds with “Losing my way a little”. The neurologist responds to this by moving on to the next question. The speech and language therapist/pathologist, who knows the client well, interjects and asks, “What do you mean by ‘losing your way a little’?”. The client explains that he has been quite depressed and having suicidal thoughts. This led to a review of the medications being prescribed. Had this been overlooked, or perhaps interpreted literally as suggesting the client was having navigational difficulties today, then this might have resulted in a significant issue not being addressed. Attending to a person’s language can be extremely helpful in deepening understanding of a person’s current perspectives, needs, and priorities. It was several clinical experiences like that outlined above, which inspired the researchers to explore metaphor use amongst people with PPA, their families and the therapists working with them.

Primary progressive aphasia (PPA) is a rare language-led dementia that typically presents between the ages of 50 and 70 years (Matias-Guiu & Garcia-Ramos, 2013). The syndrome is characterised by the gradual and pervasive decline of language function that impacts participation in daily activities, social relationships, and life roles. There are three PPA variants, each with differing language profiles (Marshall et al, 2018; Ruksenaite et al., 2021). Semantic variant PPA (svPPA) results in difficulties in using words (particularly nouns) and understanding their meanings and is associated with frontotemporal lobar degeneration. Nonfluent variant PPA (nfvPPA) results in articulatory difficulties (a motor speech disorder called apraxia) and/or difficulties in using and understanding grammar and is also associated with frontotemporal lobar degeneration. Finally, logopenic variant (lvPPA) results in difficulties in word retrieval (people report the word is on the tip of their tongue) and verbal short-term memory and is associated with Alzheimer’s disease (Gorno-Tempini et al., 2011). There are also differences in how each variant evolves, in terms of the speed and nature of progression and the stages of impairment and functional disability that individuals with PPA and their families experience over time (Hardy et al., 2023).

PPA significantly impacts psychological well-being and quality of life (Ruggero et al., 2019). Tailored supports are required to help people respond proactively to symptoms and navigate the evolving set of challenges (Volkmer, Cartwright, Ruggero et al., 2023). Research has begun to explore the healthcare experiences of people with PPA, revealing dissatisfaction with post-diagnostic services and desire to access to health professionals who understand their condition and changing needs across the continuum of care (Beales, Bates, Cartwright, & Whitworth, 2019; Davies & Howe, 2020; Ho, Cartwright, Whitworth, & Hersh, 2023; Loizidou et al., 2023). People with PPA are seeking more personalised services, as well as clearer information and direction to understand what is going on, how to deal with the practical impacts of PPA, and where to go next in order to move on beyond the diagnosis (Ho et al., 2023; Volkmer et al, 2021). In parallel to this, person-centredness has been identified as a critical element of best practice care in the PPA literature (Volkmer et al., 2023), however, this requires an intimate understanding of what PPA means to an individual and their care partners. This includes active listening to concerns and forging effective therapeutic relationships. Exploring the metaphors used by people with PPA and their supporters presents a unique opportunity to deepen understanding of how PPA is experienced and interpreted to advance care and service provision.

### Metaphor and health experience

Metaphor, when something is described using words or a phrase that symbolise or represent something else, has been studied extensively in health contexts to gain a richer and more intimate insight into health experiences (Golden et al., 2012). Metaphors are used to capture aspects of experience that are less familiar, more abstract, complex, difficult to describe, or intense (Castaño,, 2020; Golden, Whaley et al., 2012; Locock et al., 2012). Castaño (2020) highlights that metaphor use is not trivial, rather providing important insights into how a person is thinking and what aspects they deem to be most pertinent. With respect to chronic illness, individuals and their care partners often use metaphoric language to reflect ways of coping with and responding to changes in their physical functioning, communication and broader lives (Ferguson et al., 2010; Locock et al., 2012). Illness metaphors are understood to be dynamic, rather than stable; metaphors used in cancer narratives, for example, differ from use in conditions that are rare, incurable, and unpredictable, such as motor neurone disease (Locock et al., 2012). In this example, military metaphors were commonly used by people with cancer to express efforts to ‘fight the disease’, while in people with motor neurone disease they were used to express being ‘attacked by the disease’, reflecting the perceived futility of fighting a progressive neurological disease directly (Locock et al., 2012). Metaphors can indicate changes over time or responses to intervention (Levitt et al, 2000). As such, metaphors provide health professionals with a window into the emotional and personal realities of living with a chronic condition or illness, and the meaning people attach to a diagnosis and treatment, helping to facilitate more empathic and person-centred care. Health professionals can also use metaphor to support or enhance health education, comprehension and retention of information, helping to simplify complex concepts (Whaley, 2000), normalise *“what feels foreign to patients”* (Harpham, 2010, p.16), and support sense-making (Golden et al., 2012). Importantly, engaging with metaphors can help reframe experiences into a more positive light to promote wellbeing and coping responses, while strengthening trust and therapeutic alliance (Castaño, 2020; Mathieson et al., 2020).

### Metaphor and dementia

Several studies have investigated metaphor use in dementia, from the perspectives of people living with the condition and their care partners. For example, Golden and colleagues (2012) examined the metaphors used by care partners of people living with dementia. *Journey* metaphors (e.g. “going downhill”, “finding a course” or on a “two way road”) were used most frequently, followed by *machine* or *circuit* metaphors (e.g. “the system is being shut down”), *basic orientation* (e.g. *“ups and downs”), harm* or *abuse* (e.g. *“beating themselves up”), game* (e.g. *“Russian Roulette”)*, and *hand* metaphors (e.g. “reaching out”). Other examples, used less frequently, included *trap* or *prison* metaphors, *different world* metaphors, and those signifying *weight* and *struggle*. The authors provide tangible examples of how the identified metaphors could be used by nurses to help provide counselling and support, as well as to promote caregiver agency and motivate action, providing a *“rich set of linguistic tools that nurses can draw upon to make positive differences in caregivers’ lives”* (Golden et al., 2012, p.151).

In a study by Zimmerman (2017), individuals with dementia and their care partners were found to use similar metaphors. However, some, such as *journey*, were used to convey different meanings (Zimmerman, 2017). People with Alzheimer’s disease have combined the journey metaphor with the image of a labyrinth, signifying disorientation and lack of clear direction following the onset of symptoms. This conjures the image of dementia landing people in a foreign landscape that is difficult to navigate and where no return is perceived. For care partners, however, the journey metaphor was used to provide structure to their estrangement or disconnection from the person, particularly in response to communication changes (*“Ronnie’s long journey has finally taken him to a distant place where I can no longer reach him”*; Zimmerman, 2017, p.77). As such, comparing the metaphors used by people with dementia, their care partners, and other supporters may help to understand the experience of dementia from different perspectives, helping to weigh up priorities for support.

Interestingly, the term dementia has been recognised as a metaphor in itself, reflecting how society thinks about dementia (Zeilig, 2014). Unfortunately, dementia as a cultural metaphor is not a positive one, rather linked with negative stereotypes pertaining to “doom” and “a fate worse than death” (p.262), contributing to stigma and the social isolation that people with dementia experience (Zeilig, 2014). As health professionals, we have a role to play in reframing cultural metaphors and stereotypes, acknowledging that every journey with dementia is different and that people can live successfully with dementia (Love & Hummel, 2022; Wolverson et al., 2016). To our knowledge, no previous studies have explored the metaphors used to describe PPA or to consider how SLT/Ps could use metaphor to validate, educate, and empower their clients with PPA and their families, as well as to understand their own role as supporters more intimately. Further, comparing the metaphors used by people with PPA, their care partners, and SLT/Ps would help consider therapeutic alignment, also noted by Ferguson et al. (2010) in their study of metaphors used by people with aphasia after stroke, their care partners and treating SLT/Ps.

### Aims of the Study

The current study explored spontaneous metaphor use by people with PPA, their care partners, and SLT/Ps, how they are used and whether their use across these three groups aligned. We recognised from previous work on use of metaphor in illness narratives that metaphor provides a way for people to express difficult and intimate experiences and has value in sensitising health professionals’ approaches to care. In particular, we were curious about how people, even with their PPA and language loss, used metaphorical language to describe their experience. Our aims were to better understand the insights of people with PPA and their families through attending to the metaphors they use, and to understand more about the responses of SLT/Ps through their metaphorical language. We hoped to synthesise strategies, grounded in metaphor use, that would support SLT/Ps to provide more tailored, nuanced, and empathic care in partnership with people with PPA and their families.

The aims of this study were to:

- explore and compare spontaneous metaphor use by number of stakeholders (people with PPA, their care partners, and SLT/Ps)
- gain insight into the lived experiences of people with PPA
- synthesise strategies, that would support SLT/Ps to provide more tailored, nuanced, and empathic care in partnership with people with PPA and their families.

## Methods

### Design

Given the aim of this study was to examine metaphors people used spontaneously, previously collected data sets comprising naturalistic talk where metaphors were not the specific focus were examined. This is therefore a qualitative secondary data analysis which examines transcripts from two focus group studies led by the first author. The first was a study exploring the views of people with PPA and their families on what they want from speech and language therapy (Loizidou et al., 2023). The second was a study identifying best practice principles for SLT/Ps working with people with PPA (Volkmer et al., 2023). In line with previous studies exploring naturally occurring conversations for metaphor use (Jenny & Logan, 1996; Golden et al., 2012), this study employed the Metaphor Identification Procedure and analysed data extracted using an iterative narrative approach. This study was conducted in compliance with the Consolidated Criteria for Reporting Qualitative Research Checklist (Tong et al., 2007).

### Participants

#### Focus groups with people with PPA and their families

This project was part of the Rare Dementia Support (RDS) Impact Study which received approval from the UCL Research Ethics Committee (8545/004: Rare Dementia Support Impact Study). All participants consented in line with the approved procedure outlined in the RDS Impact study protocol (Brotherhood et al., 2020). Seven people with PPA and 14 CPs participated in the focus groups (see participant demographic details in Table 1). Participants were recruited via email from the Rare Dementia Support members list to participate in one of four 90-minute focus group sessions (recruitment criteria are outlined in Loizidou et al., 2023) held online via the remote conferencing. Participants were presented with the main research question: “What speech and language therapy would be most useful to you?”. Results from this study were reported by Loizidou et al. (2023).

**Table 1:** Participant demographic details

Living with PPA dataset (n=21)	
male: female	9:12
PwPPA	7
CPs	14
	(of these 5 = dyads)
CPs relationship to PwPPA (n=14)	
Wife	6
Husband	5
Daughter	3
PPA variant (dyads were counted twice for each participant)	
nfvPPA	10
svPPA	5
lvPPA	5
mixed PPA	1
Years since diagnosis	2.25 (range 1-7)
SLTs (n=15)	
male:females	0:15
Mean years as SLT/P	22.6 (range 1.5-45)
Mean no. of PPA patients seen over career clinically	141 (range 0-350+)
Mean no. of PPA patients seen over career for research	52 (range 0-200)

Notes: NB: PwPPA = people with primary progressive aphasia, CP = care partner, nfvPPA = nonfluent agrammatic variant primary progressive aphasia, svPPA = semantic variant primary progressive aphasia, lvPPA = logopenic variant primary progressive aphasia, SLT/P = speech and language therapist/pathologist

#### Focus groups with speech and language therapists/pathologists (SLT/Ps)

The study was approved by the Chairs of UCL Language and Cognition Department Ethics,

Project ID LCD-2020-14. Fifteen SLT/P participants took part in the study from Australia (*n=*5), USA (*n=*5), UK (*n=*3), and Canada (*n*=2) (see participant demographic details in Table 1). Potential participants were recruited via a snowball technique to participate in one of two 90-minute focus group meetings hosted on zoom (recruitment criteria are outlined in Volkmer et al., 2023). Focus group discussions were held to discuss the complexities of delivering interventions for people with PPA and to share opinions on best practices when working with PPA. Results from this study were reported by Volkmer et al. (2023).

## Procedures

### Transcription and data extraction

In both studies, transcriptions were automatically generated by the video-conferencing software, then edited for accuracy and anonymised. The Metaphor Identification Procedure is widely acknowledged as appropriate for the identification and analysis of metaphors in natural discourse (Gibbs, 2017) and has been used in similar research in the field of dementia (Castaño, 2020). For the purposes of this study, 100% of transcripts were read by the lead author (AV) to identify and highlight any metaphorical language used. Thorsen and Johannessen (2021) describe the boundary between literal and metaphorical language as ambiguous. In our study, Castaño’s (2020) definition of metaphorical language was used: “a more basic contemporary meaning in other contexts than the one in the given context” (Castaño, 2020, p.119; of note this encompasses similes and figurative language more broadly). On a second reading, any possible metaphorical language was highlighted and complete lexical units where this was observed extracted into a table. Transcripts were also independently analysed by LR and DH, who respectively examined 25% of focus groups with people with PPA and CPs and 25% of focus groups with SLT/Ps, extracting data into a table. Data tables were then compared for consistency, ensuring all metaphorical language had been captured by the research team. See Table 2 for an overview of the data extraction.

**Table 2:** Overview of data extraction

Stage 1	Stage 2	Stage 3	Stage 4	Stage 5
AV read the entire transcript to familiarise themselves with the content.	In the context of each lexical unit, the researcher, AV, identified if there was a more basic contemporary meaning in other contexts than the one in the given context. If yes then the text was marked as metaphorical.	Metaphorical language was extracted and collated into a table.	25% of transcripts were read by a second researcher (either LR or DH), and data extracted in accordance with stages 2 and 3.	AV compared extracted data for consistency. The research team, comprising AV, JC, LR and DH, met regularly to discuss metaphor extractions and interpretation of metaphorical language to ensure critical reflexivity.

### Analysis

To describe metaphorical themes, along with their similarities and differences, an iterative comparison method was used. This approach, associated with grounded theory, is employed by Thorsen and Johannessen (2021), and Jenny and Logan (1996). Other researchers have employed similar approaches, employing ‘parallel reading’ of data (as recommended by Steger, 2007; and employed by Thorsen & Johannessen, 2021) and meeting regularly during the analysis process to compare and discuss and come to a consensus (Locock et al, 2012; Thorsen & Johannessen, 2021). All extracted metaphorical language was coded inductively, initially into categories referring to life with PPA, caring for people with PPA or speech and language therapy by AV, DH and LR. The research team then met and discussed extracted data, identifying that much of the metaphorical language could be refined to single words or phrases. AV then charted the refined metaphorical language used by people with PPA and their care partners, to compare it directly with that used by SLT/Ps. At this point metaphorical terms were categorised into groups relating to: living with PPA, caring for someone with PPA, strategies, having speech and language therapy, access to speech and language therapy (for data from the focus groups with people with PPA) and: role of SLT/Ps, structure of speech and language therapy, delivery of therapy and therapy materials (for data from the focus groups with SLT/Ps). The research team then met and discussed emerging patterns in metaphorical language use, identifying participant diagnosis and time since onset as possible areas for further enquiry within the category of ‘living with PPA’. Metaphor use was noted to change with progression and evolution of the condition. AV then compared all metaphorical language used against participant PPA variant and time since symptom onset.

## Results

In all, 237 examples of metaphorical language were identified in the data, with 14 metaphors from people with PPA, 116 from the care partners, and 106 from SLT/Ps. Table 3 provides an overview of the number of metaphors identified and categorised into each group. A single metaphor could express more than one meaning at the same time, such that some metaphors were categorised into multiple groups following discussion with the research team.

**Table 3:** Categories and number of metaphors identified across the data sets

Data collection:	Type of metaphor (n=number of metaphors)
Data collected from people with PPA and their families - 131	Living with PPA – 45 (of these 6 were generated by PwPPA)
	Caring for a person with PPA – 37 (of these 5 were generated by PwPPA)
	Speech and language therapy – 49 (of these 2 were generated by PwPPA)
	Relationship with SLT/P - 7
	Access to services – 13 (of these 1 was generated by a PwPPA)
Data collected from SLT/Ps - 106	Delivering speech and language therapy to people with PPA - 57
	Relationships with people with PPA - 27
	Access to services - 17

Notes: NB: PPA = primary progressive aphasia SLT/P = speech and language therapists/pathologists

### Living with PPA

**Figure 1.**
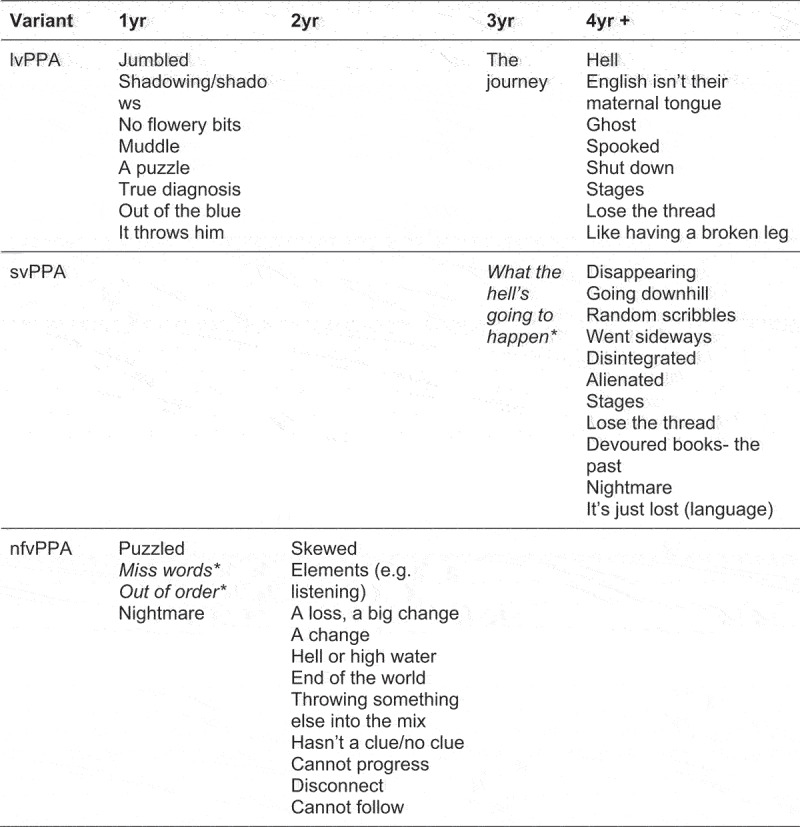
Different metaphors used by participants to describe their experiences depending on variant of PPA and time since diagnosis.

Both people with a diagnosis of PPA and their care partners used metaphorical language to reflect their experiences of living with PPA. Different metaphors were used by participants to describe their experiences depending on which variant of PPA they were living with. As shown in Figure 1, people with lvPPA and their families used metaphors about “jumbles”, “muddles” or “puzzles” conveying a sense of confusion in the first year of the disease journey e.g. “almost like a jumbled cognition going on”. The term “shadows” and “shadowing” were also used by care partners (in this case two spouses), conveying a sense that the person with PPA was judged as not fully present or participating in conversations. People with affected by lvPPA described their disease differently over time, with metaphors upgraded (becoming stronger or more emotive) as the disease progressed. People who had lived with lvPPA for more than four years invoked images through their words of “hell”, “ghosts”, and being “spooked” e.g. “she’s like a ghost behind me”.

NB: italicized and * = metaphors generated by PwPPA, vPPA = logopenic variant primary progressive aphasia, svPPA = semantic variant primary progressive aphasia, nfvPPA = nonfluent agrammatic variant primary progressive aphasia.

People with nfvPPA and their families initially used metaphors about being “missing”, “puzzled”, and “out of order” in the first year of the disease e.g. “all out of order now” These metaphors give a sense of there being some intermittent issue that needed to be worked around. Notably, “puzzle/d” was used by participants with both lvPPA and nfvPPA to express slightly different meanings. For example, “puzzled” was used to indicate a state of confusion in nfvPPA, whilst “puzzle” was used to describe a task, or specific problems encountered in lvPPA that needed to be solved. The use of metaphor by people who had been living with nfvPPA for two or three years, reflected an increase in the severity of their condition, for example, “skewed”, “disconnect”, “hasn’t got a clue”, “end of the world”, indicating a more permanent and life changing issue e.g. “they’re trying *so* hard to tell you what they want to tell you um but there’s a *huge* disconnect”.

Finally, people with svPPA and their families used metaphors such as “hell”, “alienated”, “disappeared”, “disintegrated”, and “nightmare” e.g. “her vocabulary was disappearing”. These metaphors were collected from people who had been diagnosed at least 3 years prior, conveying a deep sense of loss and complete change in their lives.

#### Caring for someone with PPA

Care partners of people with PPA described their own experiences using metaphorical language. Despite describing themselves as experiencing similar things to the person with PPA – being “in the same boat” as their partners – they felt they held more responsibility as the “guide along with it”. Carers described the sense of being rather unprepared for this journey where they had to seek out how best to manage through “discovery”, whereby “finding out” required them to “come at it from a different angle”. Some described a visual search, such as “looking for” something, whereas others mentioned epiphanies (“it dawned on me”).

This journey was difficult. Care partners described tension in their role “feeling very torn” and pressured to perform as a carer; “I had to get it right”. There was a sense of simply surviving: “get through”, and sometimes some things could not be resolved and therefore “parked” or “let go”. For some it was an unfeasible challenge to “tame that demon”. For others, they felt they had been put on trial; “guilty” and “guilty as charged”.

The carers sought to establish order by “dealing with the muddle”, identifying “a system”, finding “space to process” or finding “the right track”. They explained the motivation for using specific strategies as a way of reducing the risk of failure and to facilitate function and independence, for example, “you don’t want the person to constantly fall over”. Importantly, these strategies needed to be timely and targeted (e.g., “snappy”) to succeed. Success, however, was not guaranteed and strategies could also have a negative impact, for example, they could “throw you completely” or become “a nightmare”. Some strategies were considered quite unfeasible to use; “never on the radar” or “not a natural medium”.

##### SLT/Ps

SLT/Ps identified their role as overseeing a “toolbag”, “toolkit”, or a “building” of therapeutic options. These intervention options might be drawn from an array of “buckets”, “layers”, “silos”, “tiers”, “levels”, or “steps”, all of which might be drawn upon for different people at different points in their disease progression; “growing”, and “evolving”. SLT/Ps described services for PPA as having multiple component parts that needed to be “unpacked”, “broken up”, “tweaked” “sought”, “fine grained”, delivered in a complex method through “hybrid” approaches or a “buffet”. Despite the complexity of the SLT/P’s oversight, however, they described their role as creating clarity and structure by providing a “roadmap” or “umbrella” for people with PPA and their families.

### Relationships between people with PPA and their care partners and the SLT/Ps

People with PPA and their families and SLT/Ps used metaphors to describe their relationships with one another. People with PPA and their families described the therapist as a “guide” who could “provide any kind of fix”. They felt this professional could be “a person who’s outside and can look in”. Similarly, SLT/Ps described themselves “walking alongside” the person and their family, trying to provide some structure or stability using actions to describe this such as “build” or “cement”. Other people with PPA and their families felt there was something more elusive or intangible about the therapist, that they could provide “some magic”.

Not all people with PPA and their families were so certain about their relationship with the SLT/P, however. Some described uncertainty: “it’s rather like going into a chocolate shop blindfolded. You don’t know what there is to pick from”. One person described having “lost faith” in speech and language therapy whilst another recognised that perhaps what they wanted was unattainable, “asking for the moon”. Considering that speech and language therapy ultimately cannot slow disease progression, families are not necessarily going to find relief, whatever therapy choices are made.

SLT/Ps also identified that their relationships with people with PPA and their families could be complex, describing them as “delicate” and a “difficult line to tread”. This could feel relentless such that SLT/Ps described relationships as “draining” or that they felt it was “constant wound after wound you are nursing them through”.

### Access to speech and language therapy

People with PPA and their families described speech and language therapy as a part of their journey with PPA. For example, they described speech and language therapy as something you “go through at the beginning of the journey”, a place they returned to at different points in time (e.g. “we have a way back”), or something that was no longer needed (e.g. “no need to go further”). They also described how speech and language therapy was not always available where “the door’s been closed”. This was often due to external forces, “it depends on how the purse holders decide how to slice the cake”, where funding or services had “dried up” or “been decimated”.

SLT/Ps also described themselves as dealing with similar external forces, for example: “we are fighting for PPA” and the work is a “revolt”. They often used metaphorical language to indicate a lack of control over external constraints meaning that some people “fall in the cracks”, whilst for others it is “potluck” if they access speech and language therapy. SLT/Ps expressed concerns over serious negative consequences and that poor access could be “a real tragedy”.

## Discussion

This is the first study to explore the use of metaphorical language by people with PPA, their families and treating SLT/Ps. As found previously in relation to both aphasia (Ferguson et al., 2010) and dementia (Golden et al., 2012), the way people use language provides insights into their experiences and perspectives that transcends the literal words produced. While it could be assumed that the language impairment in PPA would reduce the value of this exercise, our findings suggest that listening to the language used by people with PPA and their care partners is clinically useful to deepen understanding of how they are living with PPA, as well as how their experiences might vary across variants and over time. The language examples show the sense of confusion, the hell, and the alienation of PPA in a way that could be otherwise hard for people to convey. Finally, we identified differences in the way SLT/Ps spoke about PPA, when compared to those living with PPA and their care partners, with implications for therapeutic alignment and relationships. We will explore the findings below, culminating in clinical implications and consideration of ways to engage with metaphor in practice to extend understanding of PPA, appreciation of the SLT/P role, and capacity to provide more deeply connected and responsive care.

There has been growing interest in clinical staging systems for PPA that provide a clear roadmap for individuals living with PPA, their families, and other supporters (Hardy et al., 2023; Hinshelwood et al., 2016). Importantly, health professionals, including SLT/Ps, find the differential diagnosis of PPA challenging, and prognostic conversations have been difficult without a reliable means of anticipating progression of symptoms over time (Hardy et al., 2023). In the current study, subtle differences in use of metaphorical language across the PPA variants, suggested there is an embodied reality of those variants for people who live with them and for their close family members. This gives differential diagnosis between PPA variants some validation in terms of perceptions of loss. For example, in lvPPA, references to “ghosts”, “shadows”, and being “spooked”, conjured a different experience to that of the metaphors of svPPA such as “disintegration”, “alienation”, and “disappearing”. The former are suggestive of the person being just out of reach (the person may know what they want to say but unable to quite grasp it in lvPPA), whilst the latter indicate they have gone (the semantic knowledge has deteriorated in svPPA). Examining how metaphorical language differs and evolves has potential to inform staging systems and further qualify the stages of impairment and functional disability that people move through or experience over time (Hardy et al., 2023). Importantly in this study, for people with PPA, metaphoric language was present and powerful but not plentiful. As people’s language becomes more impaired, use of metaphor to express themselves may become less prominent, however, it is important SLT/Ps continue to listen to the person and their families. Through metaphorical understanding, we may be able to help people anticipate the future, support more nuanced and proactive advance care planning, and make sense of experiences at different points of each person’s journey. Ultimately, metaphorical understanding could help promote empathic staging systems, as part of person-centred care for people with PPA.

This work builds on previous research undertaken by Golden et al. (2012) and Zimmerman (2017) who identified common metaphors used by people with dementia and their care partners highlighting that people use metaphors to describe abstract experiences that are difficult to explain verbally. Metaphors used by people with Alzheimer’s and their families observed in social media have some similarities with the data collected in this study. Dementia has been described as a ‘thief’ in poems published on social media platforms (Hillier, 2018, https://www.facebook.com/HillierPoetry), or a person with Alzheimer’s disease described feeling they were ‘drifting off the earth’ (https://www.youtube.com/watch?v=uAlkCMfTASQ). The latter example overlaps closely with the ghostly metaphors used by people with lvPPA in this study. The similarities in the pathological basis between Alzheimer’s disease and lvPPA may account for the overlapping and difficult-to-describe experiences conveyed by these metaphors.

Whilst there is research demonstrating changes in social cognition in people with svPPA and nfvPPA (Fittipaldi et al., 2019), which will inevitably affect their ability to understand how other people see the world, this study focuses more on a person’s own experiences and fears. This study therefore indicates that there may be a disparity between a person’s ability to read others’ emotions, versus conveying their own when living with a PPA. Moreover, the embodied reality of living with different types of dementia conveyed by the metaphors used in this study highlights the value of active and empathic listening by the professionals around the person, throughout the diagnostic process.

SLT/Ps in this sample did not use metaphors to describe PPA itself, rather their metaphors reflected how they approached management and responded to PPA, regardless of the variant. Whilst the purpose of the original focus groups with the SLT/Ps was not to describe PPA, and thus may account for this difference in metaphor use, there were also some similarities between data sets. Both SLT/Ps and care partners expressed a need to find some order. Some care partners identified SLT/Ps as facilitators of this, who could relieve some of the load for them while others found SLT/P services ineffectual or difficult to access. It is possible that SLT/Ps who are unsure what to do or are unable to offer a service are perceived as having “closed their doors”. SLT/Ps in our study recognised this, using similar metaphorical language to describe the devastating effects of when people were unable to access speech and language therapy. Despite these service-level limitations, the impact of PPA increases over time, and SLT/Ps’ metaphoric language reflects their discomfort with this growing challenge, with a (perhaps more medicalised) dosage-based approach, rather than a person-centred or relationship-focused approach (Volkmer et al., 2023).

The findings from this study highlight the important role that SLT/Ps should be taking in ensuring people with PPA have *a rope team*, a metaphor previously used in the stroke aphasia literature (Worrall, 2019). People with PPA must be connected with the right supports at the right time, provided with accessible information to make sense of the diagnosis, and provided with strategies to stay connected, as “part of this world”, rather than alienated as symptoms evolve (Volkmer et al., 2023). Gaining a deeper understanding of the experience and emotional needs of people with PPA and their families, and who they need to connect to along the journey of care, are key recommendations from the best practice principles for SLT/Ps working with people with PPA (Volkmer et al., 2023). Studying the words of small numbers of people in depth highlights the value of listening to *how* people tell their stories. Attending to social and mainstream media stories, and the language used in these contexts, is also valuable. In 2022, the high-profile case of actor Bruce Willis revealed the burden of the disease for him and his family (https://www.theguardian.com/film/2023/feb/16/bruce-willis-frontotemporal-dementia-aphasia-ftd). Until now there has been no cultural metaphor or reference point for PPA. The unfolding description of Bruce Willis’ “changing brain”, as his wife put it, serves as further evidence for the complex journey people travel. We need to be more attuned to their experience and acknowledge that people use metaphor to talk about things that are complex or difficult to describe (Castaño, 2020). Subtle attention to language can help SLT/Ps to ‘reframe’ thinking or maintain hope (Bryden, 2018), and can help audit and evaluate services to avoid a sense of disconnection and closing doors.

### Clinical implications

Responding to metaphor has potential to deepen understanding and strengthen therapeutic relationships, promoting trust and connectedness, as well as helping people feel less isolated and better supported. This paper does not describe how to formally assess a person’s ability to use or understand metaphors in a clinical setting; we only advocate ‘listening out for them’ during conversations in order to respond to them. Based on the results of this study, therapists may find it useful to take account of metaphors used by either the person or their care partner, such as “jumbles”, “spooks” and “shadows” when considering a diagnosis of lvPPA, “missing”, “skewed” or “out of order” when considering a diagnosis of nfvPPA and “alien”, “nightmare” or “hell” when considering a diagnosis of svPPA.PPA threatens self-expression and this may mean that hearing people’s experience of living with the disease becomes harder in time. Disease progression may even be seen in the decline of metaphor use in an individual. Making a note of whether metaphors use by care partners is upgraded or becomes stronger over time e.g. transitioning from “shadows” to “ghosts” over several months or years of knowing a person with lvPPA, may be helpful for signposting disease progression

Listening out for metaphors requires therapists to use reflective listening skills and delve deeper into feelings and thoughts that may have only been alluded to in metaphorical form, for example, *“When you say he’s like a ghost what do you mean?”* (Mathieson et al., 2020). Having key metaphors in the back of mind when working with people with PPA and their care partners allows for improved, and shared, decision making. The ‘chocolate shop’ of speech and language therapy, as it were, needs to be fully stocked and well signposted. People with PPA and their families may think something enticing (helpful) is there but may not be able to see it or grasp it without support. In fact, people with PPA and their families may expect more from speech and language therapy than is often available, for example improvement or resolution of speech and language difficulties. By explaining all the evidence-based therapy options available, we can support people to better understand the role of the SLT/Ps and ‘see’ the options available.

**Figure 2.**
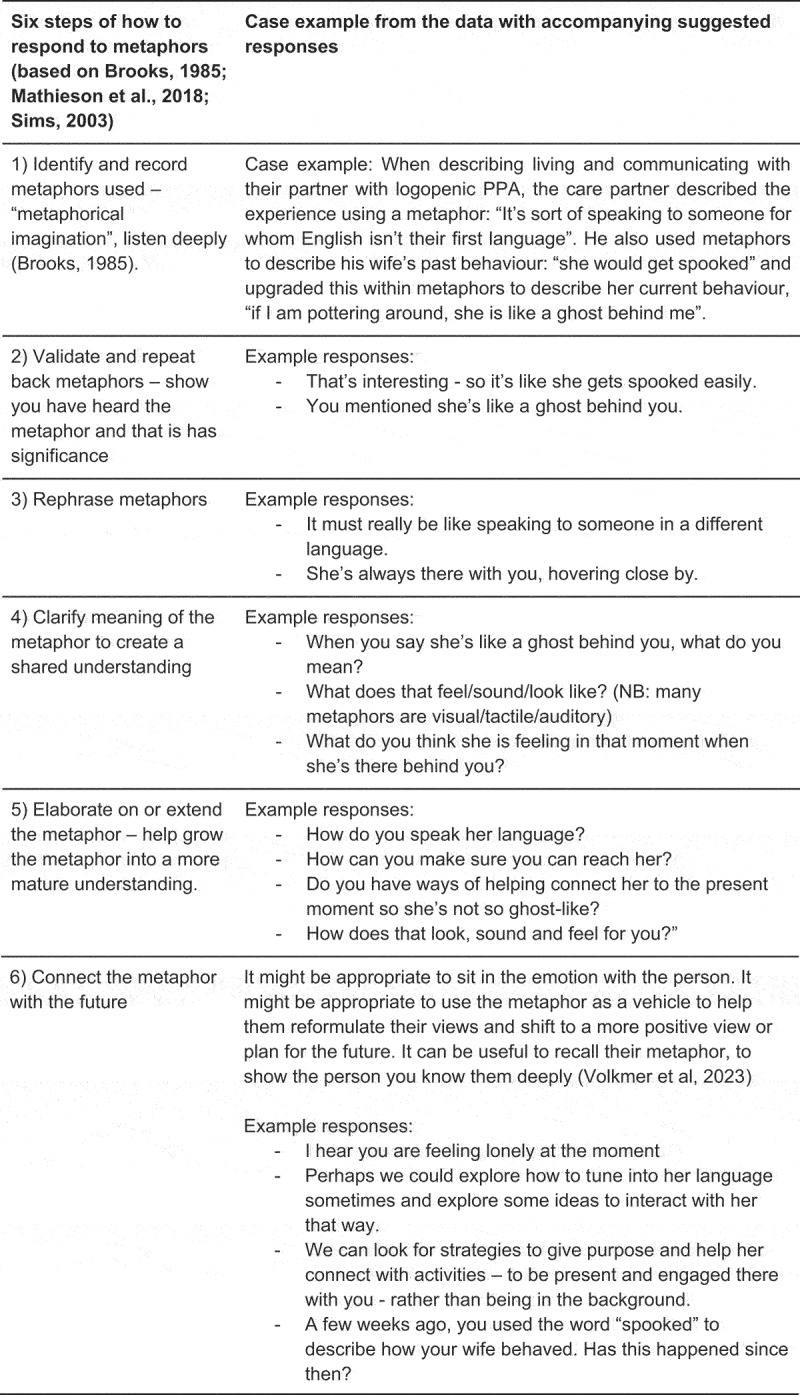
Case example accompanied with suggested responses using six-steps to illustrate how to respond to metaphorical language

SLT/Ps and other healthcare professionals need to consider how to develop their metaphorical competence. Research on responding to metaphors from the psychotherapy, cognitive behavioural therapy and stroke aphasia literature (Brooks, 1985; Mathieson et al., 2020; Sims, 2003; Lanzi et al., 2021) was consulted to address the third aim of this study; to synthesise strategies, that would support SLT/Ps to provide more tailored, nuanced, and empathic care in partnership with people with PPA and their families. Metaphorical competence requires the SLT/P to have “metaphorical skill” (Brooks, 1985) – to be able to listen out for, comprehend and respond to metaphors produced – to identify the most important metaphors, for example the ones that recur and that appear most salient and insightful for that person at that point in time. Responding to metaphors also requires “metaphorical imagination”, whereby we bend our own world view and perspectives and remain open to other ways of seeing a problem or situation (Brooks, 1985). AV selected a participant at random from the transcript. All the metaphors and surrounding text used by this participant were identified in the transcript and AV, JC, LR and DH discussed responses to these metaphors. Consequently, the same approach was applied to two further participants. From this discussion, AV and JC synthesised a table, providing an overview of responses and steps that could be taken. This was circulated to AV, JC, LR and DH and consequently finalised. Figure 2 presents a specific case example from the data, with the proposed accompanying series of steps illustrating the clinical application of how SLT/Ps could respond to metaphors in a therapeutic interaction (based on Brooks, 1985; Mathieson et al., 2020; Sims, 2003; Lanzi et al., 2021).

NB: PPA = primary progressive aphasia

### Limitations and future directions

The small number of metaphors collected from people with PPA (14) is likely attributable to the small number of people with PPA (7) compared to care partners (14) who participated in this study. Moreover, the focus group format resulted in less contributions from people with PPA than the care partners, meaning there was less opportunity for people with PPA to use metaphors. Future research on metaphor use in PPA should focus on a larger pool of participants and make provision for more equitable numbers and thus opportunities for contributions. Larger sample sizes would also benefit a more balanced representation from across the disease variants and time post onset of symptoms in order to lend more confidence to the tentative patterns in metaphor use observed in this study. Despite this limitation, the use of naturalistic data reinforces that metaphors are a genuine and authentic part of how people with PPA, their families and SLT/Ps describe their experiences. Golden and colleagues (2012) also provide interesting next steps to their research - for example, presenting metaphors identified back to participants, then asking which ones they rate as most helpful or comforting or reassuring or which ones help their understanding of the condition. Alternatively, audio or video recording a series of therapy sessions within a single case study and exploring the use of metaphors over time would also be useful. These novel ideas could assist in co-designing how metaphors could be used in clinical care, counselling and education in PPA to ensure they meet the needs of people living with this diagnosis.

It would also be interesting and valuable to understand how people with PPA understand and use metaphors over time. Metaphors are often abstract concepts; one might expect people with svPPA to be least able to use these. Indeed, only a very small number of metaphors in this study were generated by people with PPA. Future research should explore whether use of metaphor varies across PPA variants in terms of mental (compared to physical) content and for non-literal (compared to literal) referents. Another limitation of this study is that a full range of socio-demographic characteristics of participants was not collected. The people who participated in this study were from English speaking backgrounds and based in the UK, meaning that the observations around metaphorical language may be culturally skewed. Future research needs to explore the use of metaphors from different linguistic, cultural and socio-demographic backgrounds to enhance our understanding of what people with PPA and their families and the health and social care professionals can do to support them when living with PPA. Comparing metaphors generated by participants across studies might be one way to facilitate this type of analysis in future studies.

## Conclusions

People with PPA, their families and SLT/Ps use metaphorical language to convey their experiences. These metaphors change over time and can vary across different PPA variants. Although SLT/Ps also use metaphors, these are predominantly related to how to structure support. SLT/Ps can attend to the metaphorical language used by people with PPA and their families to encourage alignment and to see beyond the label of PPA to recognise the individual’s support needs. SLT/Ps and other health professionals can add listening to, and acting on, metaphoric language to their management, at all points along their patient’s journeys with PPA.
